# Comparison of Magnetic Resonance Imaging Features Between Programmed Cell Death 1/Programmed Cell Death Ligand 1 Inhibitor-Induced Hypopituitarism and Idiopathic Hypopituitarism in Japanese Subjects

**DOI:** 10.7759/cureus.71699

**Published:** 2024-10-17

**Authors:** Yuichiro Iwamoto, Tomohiko Kimura, Kazunori Dan, Hideyuki Iwamoto, Junpei Sanada, Yoshiro Fushimi, Yukino Katakura, Masashi Shimoda, Shuhei Nakanishi, Tomoatsu Mune, Kohei Kaku, Hideaki Kaneto

**Affiliations:** 1 Department of Diabetes, Endocrinology and Metabolism, Kawasaki Medical School, Kurashiki, JPN

**Keywords:** hypopituitarism, idiopathic hypopituitarism, immune checkpoint inhibitors (icis), immune-related adverse events (irae), magnetic resonance imaging (mri)

## Abstract

Objective

Immune checkpoint inhibitor-associated hypopituitarism (ICI-HP) is a rare immune-related adverse event (irAE) of ICIs. This study aimed to identify characteristic pituitary findings on magnetic resonance imaging (MRI) in ICI-HP.

Methods

This study is a single-center, retrospective, observational study. Among 95 patients admitted to our hospital for examination of hypopituitarism between January 2010 and December 2022, 26 patients with normal pituitary function (NPF), 13 patients with ICI-HP, and 13 patients with idiopathic hypopituitarism (IHP) were included and analyzed.

Results

For participants with ICI-HP, the time from ICI administration to ICI-HP diagnosis was 125 (56-212) days. Compared to the NPF, participants in the ICI-HP and IHP were significantly older (p<0.01) - the highest number of patients presented with isolated adrenocorticotropic hormone deficiency in both ICI-HP and IHP. Pituitary volume was not significantly different among the three groups (p=0.68). The width of the pituitary stalk was 1.8 (1.5-2.0) mm in NPF and 1.8 (1.7-2.2) mm in IHP, whereas it was 2.4 (2.4-2.7) mm in ICI-HP and was significantly larger compared to the other groups (p<0.01).

Conclusion

This study identified no pituitary enlargement in ICI-HP compared to NPF or IHP. However, the pituitary stalk was significantly thickened in ICI-HP compared to the other groups. Pituitary stalk enlargement may be a characteristic MRI finding of ICI-HP.

## Introduction

The anterior pituitary gland regulates the functions of many peripheral endocrine tissues, including the thyroid, adrenal gland, and gonads. Hypopituitarism causes hormone deficiency symptoms in peripheral tissues, necessitating endocrine replacement therapy [1]. Most hypopituitarism is neoplastic in origin, but inflammation and drug-induced hypopituitarism may also occur [2]. Immune checkpoint inhibitors (ICIs) disrupt many endocrine tissues, including the thyroid, adrenal, and pituitary glands [3]. Pituitary inflammation caused by cytotoxic T lymphocyte-associated antigen-4 (CTLA-4) inhibitors and programmed death-1 (PD-1) inhibitors was first reported in 2003 [4]. Pituitary-related immune-related adverse events (irAE) are known to occur as frequently as 3.2% after ICI administration [5,6]. ICI-induced hypopituitarism (ICI-HP) is often irreversible, requiring hormone replacement in more than 89% of patients, with a reported mortality rate of 2% [7,8]. Although our knowledge of the histopathologic findings of ICI-HP is inadequate, postmortem examination of cases of ICI-HP suggests the involvement of lymphocytic and necrotizing hypophysitis in the pathology [8]. On the other hand, the imaging features of hypopituitarism caused by ICI are not established [9]. The purpose of this study was to clarify the characteristics of pituitary MRI images in patients with ICI-HP by comparing them with patients with normal pituitary function.

The results of this study were reported at the 35th Meeting of the Society for the Study of the Interbrain, Pituitary, and Adrenal System.

## Materials and methods

Study population and patient preparation

**Figure 1:**
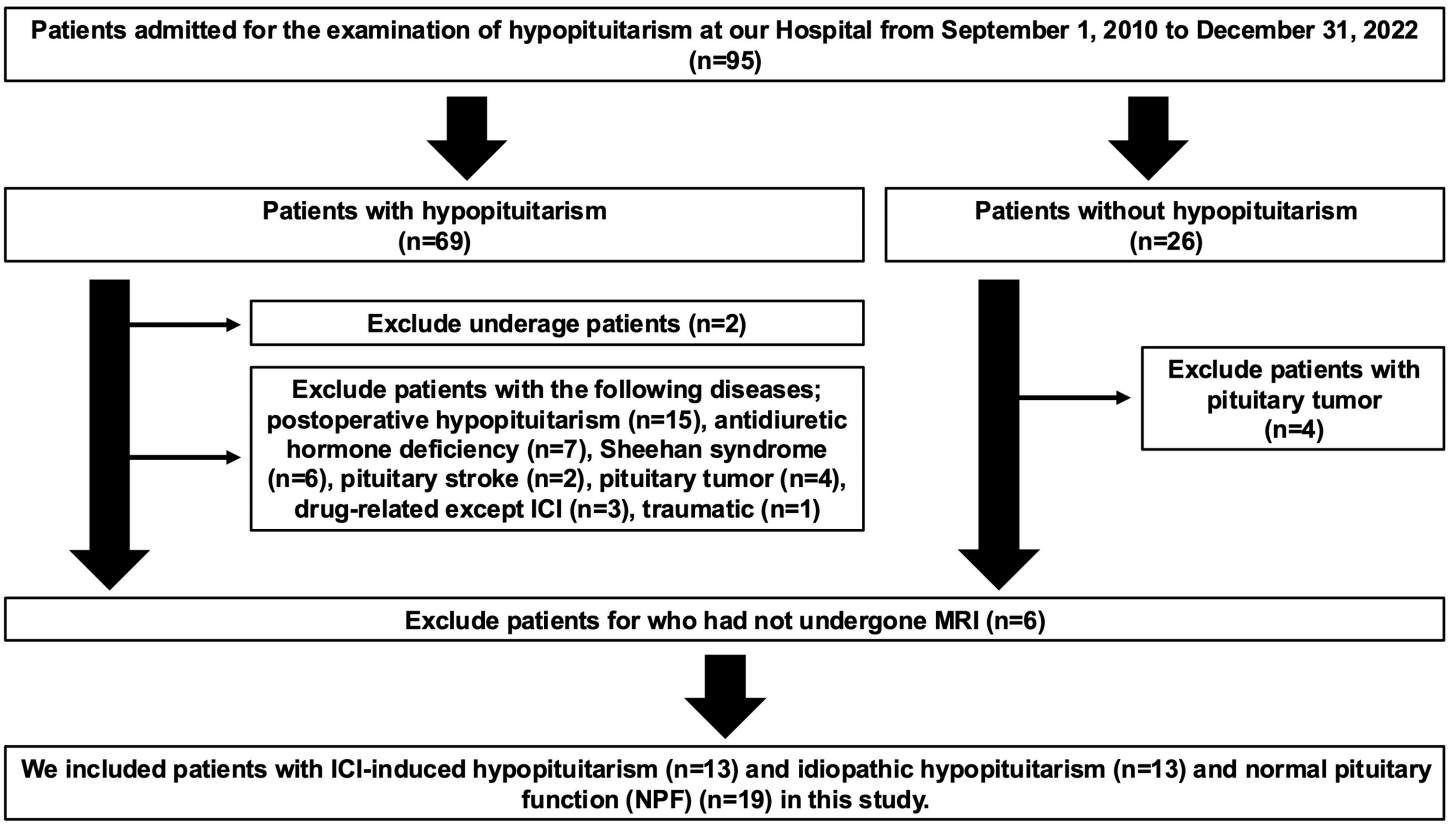
A flowchart of the participants in this study ICI: immune checkpoint inhibitor

This is a single-center retrospective observational study conducted at Kawasaki Medical School Hospital. The Institutional Review Board of Kawasaki Medical School approved this study, and opt-out informed consent was obtained from the study subjects (No. 5991-00). A flowchart of the participants in this study is shown in Figure 1. Among the patients admitted to the Department of Diabetes Metabolism and Endocrinology, Kawasaki Medical School Hospital, from January 1, 2010, to December 31, 2022, 69 patients were diagnosed with hypopituitarism. Among the patients diagnosed with anterior hypopituitarism, there were underage cases (n=2), postoperative hypopituitarism (n=15), antidiuretic hormone (ADH) deficiency (n=7), Sheehan syndrome (n=6), pituitary stroke (n=2), pituitary tumor (n=4), drug-related except ICI (n=3), traumatic (n= 1), and patients who had not yet undergone pituitary MRI (n=3) were excluded from the study. Finally, 13 subjects with ICI-HP and 13 subjects with idiopathic hypopituitarism (IHP) were included in the study. In addition, 26 patients were diagnosed with normal pituitary function (NPF) by endocrine testing during hospitalization. Patients with pituitary tumors (n=4) and no pituitary MRI (n=3) were excluded from this study, and 19 subjects with NPF were included in the analysis.

Diagnosis of ICI-induced hypopituitarism

As explained in the previous section, patients with hypopituitarism of the posterior pituitary were not included in the study while patients with hypopituitarism of the anterior pituitary were included. This is because the subjects with ICI-induced hypopituitarism experienced at our hospital did not include any patients with hypopituitarism of the posterior pituitary gland. In our hospital, the growth hormone-releasing peptide-2 (GHRP2) test and corticotropin-releasing hormone (CRH)-luteinizing hormone-releasing hormone (LHRH)-thyrotropin-releasing hormone (TRH)-stimulating test were performed to diagnose hypopituitarism, and hypopituitarism was diagnosed when there was hypopituitarism in any of the anterior pituitary lobes. In the GHRP2 test, growth hormone (GH) secretion was judged to be low if the peak GH level was 9 ng/mL or less before and 60 minutes after GHRP2 loading. The CRH-LHRH-TRH-stimulating test was started 30 minutes after waking up and after resting. If the ACTH peak value after the intravenous administration of CRH 100 μg was less than 1.5 times or less than 30 pg/mL, ACTH deficiency was diagnosed. If the peak LH and FSH levels after intravenous administration of 100 μg LHRH were less than twice the normal levels, and if the peak LH and FSH levels 30 minutes after LHRH loading were less than twice the normal levels, the patient was diagnosed with LH and FSH deficiency. If the peak TSH level 30-60 minutes after intravenous administration of 500 μg TRH was less than 10 μU/mL, the patient was diagnosed with TSH deficiency. Although the participants in this study did not include patients who were using steroid preparations at the time of the examination, three of the ICI-HP cases had a history of steroid treatment for the purpose of treating malignant tumors.

Methods of evaluating pituitary morphology by MRI

**Figure 2:**
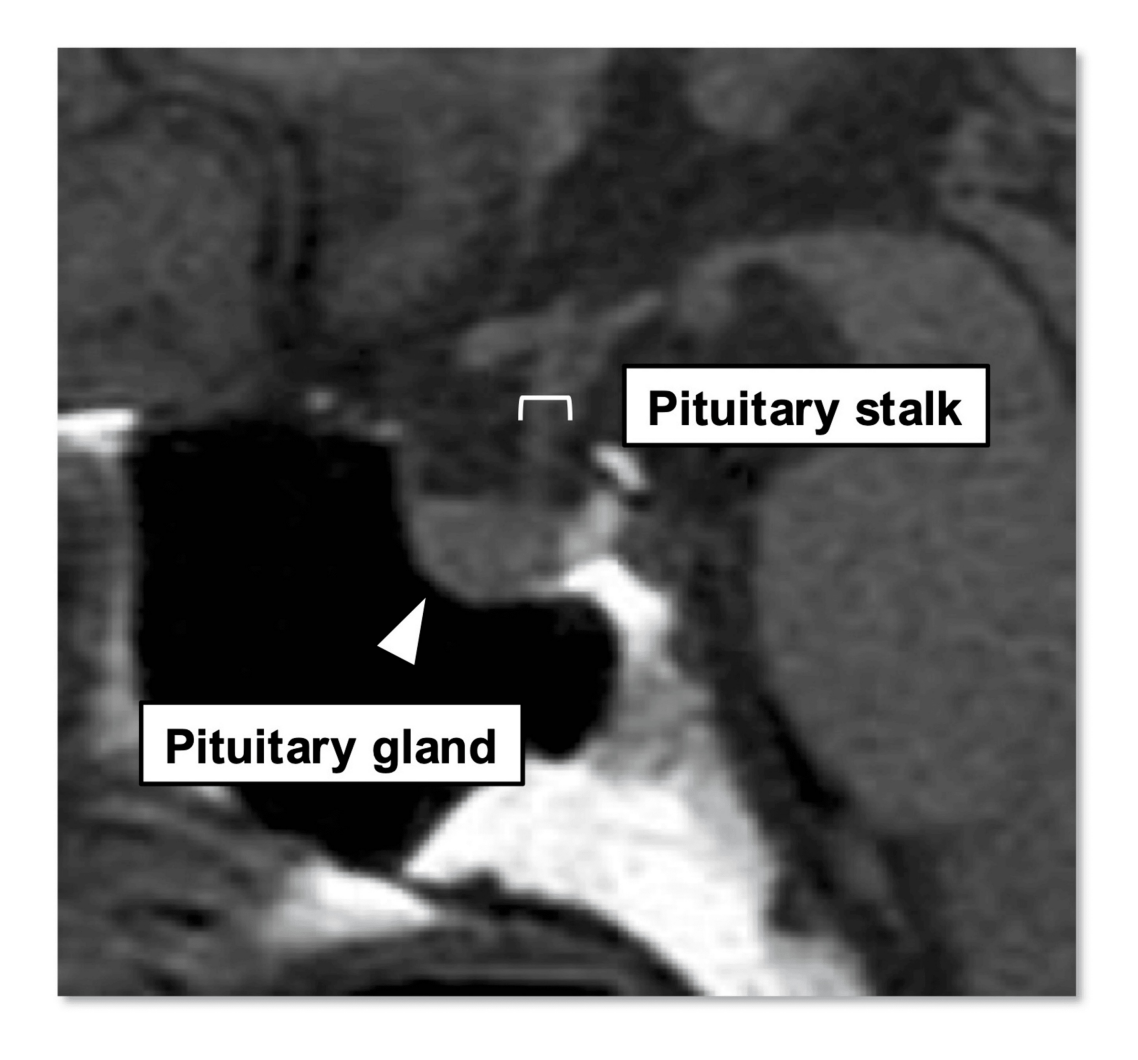
Measurement sites on MRI of the pituitary gland

The pituitary volume and width of the pituitary stalk were measured from MRIs taken during hospitalization (Figure 2). All participants had their pituitary morphology assessed using a 3-Tesla MRI. SYNAPSE VINCENT (FUJIFILM, Tokyo, Japan) was used to measure pituitary volume and stalk width. Pituitary volume was calculated using the ellipsoid method, measuring the maximum cross-sectional area of the sagittal section and the height of the horizontal section [10]. The width of the pituitary stalk was determined by measuring the upper and lower pituitary stalks in the sagittal section at two locations and averaging the results.

Statistical analysis

Data are expressed as median (interquartile range). The primary endpoint is to identify characteristic MRI findings in ICI-HP. Kruskal-Wallis and chi-square tests were used to assess parameter differences among the three groups. The Dunn test was used as a post hoc test. The Wilcoxon test was used as a two-group test for ICI-HP and IHP combined with IAD. To evaluate the effect size, eta-squared was calculated for the Kruskal-Wallis test and r for the Wilcoxon test. JMP Pro version 17.0.0 (SAS Institute Inc., North Carolina, USA) was used for all statistical analyses. Microsoft Excel for Mac version 16.87 (Microsoft Corporation, Redmond, WA, USA) was used to create tables and figures.

## Results

Clinical characteristics of the participants in this study

The clinical characteristics of the participants in this study are shown in Tables 1, 2. Compared to the NPF, participants in ICI-HP and IHP were significantly older (p<0.01). Body weight, vital signs, and electrolytes at admission did not differ (Table 1). The diagnosis of clinical endocrine disorders in ICI-HP and IHP is shown in Table 2; the highest number of patients presented with isolated adrenocorticotropic hormone deficiency (IAD) in both ICI-HP and IHP. Among ICI-HP participants, the ICIs used were nivolumab in 7 cases (43.8%), pembrolizumab in 6 cases (37.5%), atezolizumab in 2 cases (12.5%), and avelumab in 1 case (6.3%). Malignancies among ICI-HP participants include non-small cell lung cancer in three instances, malignant melanoma, and gastrointestinal and renal cell carcinoma in two cases each, and hepatocellular carcinoma, gingival carcinoma, and cancer of unknown primary each in one case. For participants with ICI-HP, the time from ICI administration to diagnosis of ICI-HP was 125 (56-212) days. Table 3 shows the chief complaint at admission for each group. The most common chief complaint in each group was general malaise, with ICI-HP and IHP having a higher percentage of decrease in appetite as the chief complaint as compared to NPF (p=0.0029). Headache was the only complaint in NPF cases (p=0.050).

**Table 1:** Clinical parameters of the participants of this study Data are presented as median (IQR). Kruskal-Wallis test and chi-square test were used for analysis. NPF, normal pituitary function; ICI-HP, immune checkpoint inhibitor-induced hypopituitarism; IHP, idiopathic hypopituitarism; BMI, body mass index

Parameters	NPF (n=19)	ICI-HP (n=13)	IHP (n=13)	chi-squared approximation	p-value
Male/female	14 / 5	10 / 3	10 / 3	9.23	<0.01
Age (years)	47 (33-56)	66 (57-69)	67 (60-74)	18.42	<0.01
Height (cm)	159 (153-164)	167 (157-169)	162 (160-169)	2.30	0.18
Body weight (kg)	57 (48-66)	61 (53-70)	53 (45-66)	2.11	0.46
BMI (kg/m^2^)	23 (19-28)	23 (20-26)	20 (17-25)	1.85	0.27
Systolic blood pressure (mmHg)	125 (105-143)	129 (112-147)	116 (102-132)	1.26	0.32
Diastolic blood pressure (mmHg)	71 (62-85)	74 (63-86)	66 (62-77)	0.28	0.54
Pulse rate (beats per minute)	75 (67-83)	82 (66-90)	76 (67-94)	0.22	0.90
Body temperature (℃)	36.5 (36.3-36.7)	36.6 (36.2-36.9)	36.5 (36.3-36.8)	0.20	0.97
Blood glucose (mg/dL)	95 (91-105)	98 (85-127)	82 (72-99)	1.44	0.21
HbA1c (%)	5.9 (5.4-6.6)	5.9 (5.6-6.2)	5.3 (5.0-5.8)	3.56	0.14
Serum sodium (mmol/L)	140 (139-142)	139 (137-143)	138 (133-143)	0.35	0.51
Serum potassium (mmol/L)	4.2 (3.7-4.3)	4.3 (3.6-4.7)	4.2 (4.1-4.6)	1.52	0.29
Serum chloride (mmol/L)	104 (102-106)	103 (100-106)	102 (101-107)	0.56	0.65

**Table 2:** Diagnosis of clinical endocrine disorders in patients with ICI-HP and IHP ICI-HP, immune checkpoint inhibitor-induced hypopituitarism; IHP, idiopathic hypopituitarism; IAD, isolated adrenocorticotropic hormone deficiency; ITD, isolated thyroid-stimulating hormone deficiency; ACTH, adrenocorticotropic hormone; TSH, thyroid-stimulating hormone

Diagnosis	ICI-HP	IHP
IAD, n	11	9
ITD, n	2	1
ACTH, TSH secretory deficiency, n	1	0
Anterior pituitary hypopituitarism, n	0	3

**Table 3:** Study participants' chief complaint at admission The chi-square test was used for analysis. NPF, normal pituitary function; ICI-HP, immune checkpoint inhibitor-induced hypopituitarism; IHP, idiopathic hypopituitarism; ACTH, adrenocorticotropic hormone

Diagnosis	NPF	ICI-HP	IHP	chi-squared approximation	p-value
General malaise, n	10	8	7	0.27	0.27
Decrease in appetite, n	1	7	7	13.56	<0.01
Headache, n	4	0	0	7.44	0.050
Hypoglycemia, n	1	2	1	0.95	0.60
Disturbance of consciousness, n	0	1	2	3.83	0.23
ACTH elevated, n	3	0	0	5.47	0.11
High fever, n	1	1	0	0.48	0.62
Others, n	Mood disorder, 1, Amenorrhoea, 1	0	Hand tremor, 1	0.42	0.51

Pituitary findings on MRI

**Figure 3:**
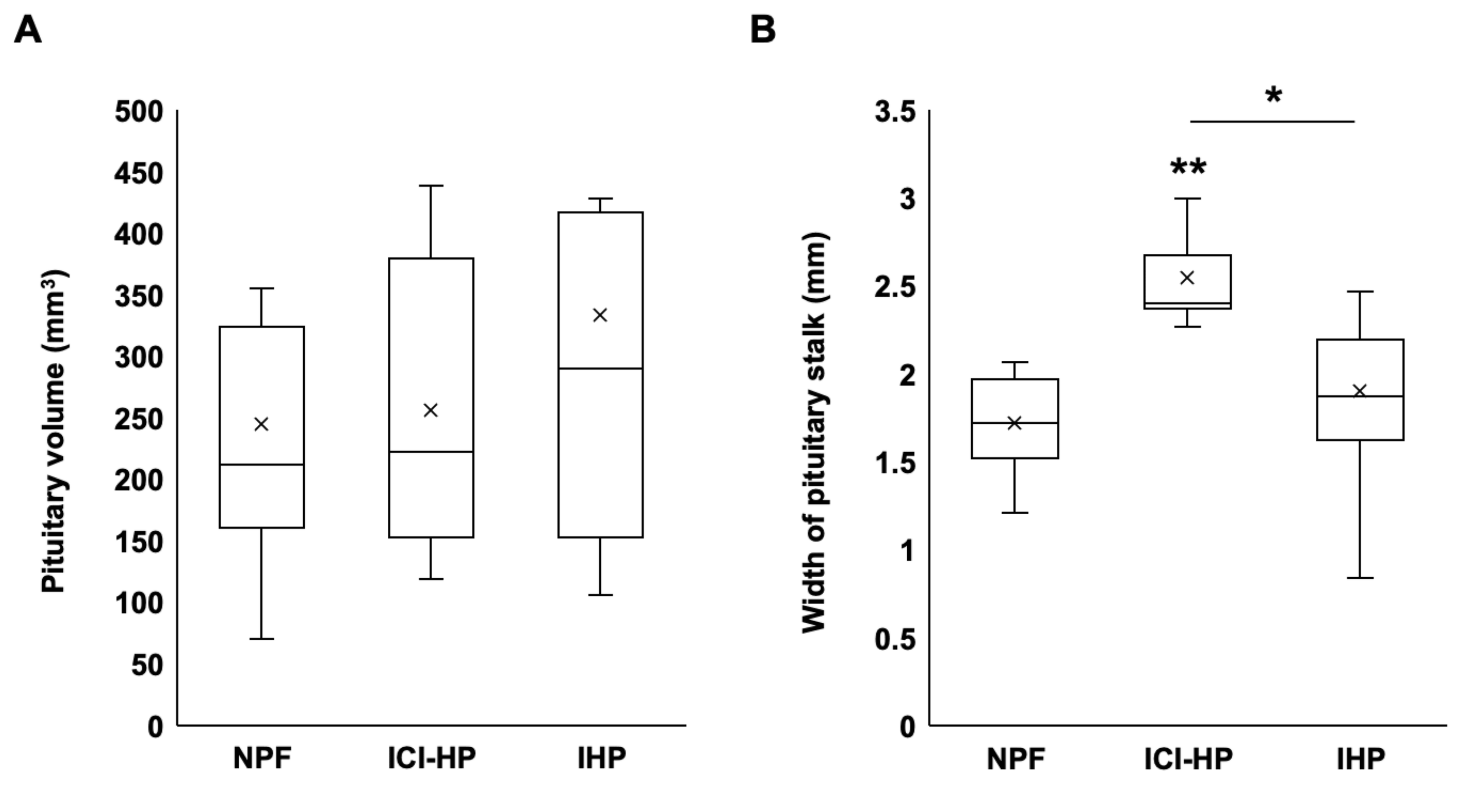
Pituitary findings on MRI Pituitary findings on MRI in normal pituitary function (NPF, n=19), immune checkpoint inhibitor-induced hypopituitarism (ICI-HP, n=13), and idiopathic hypopituitarism (IHP, n=13). (A) Pituitary volume (p=0.68, eta-squared=-0.03). (B) Pituitary stalk width (p<0.01, eta-squared=0.455). The Kruskal-Wallis test was used for the analysis, and the Dunn test was used as a post hoc test. * p<0.05, ** p<0.01.

The differences in MRI findings of the pituitary gland in each group are shown in Figure 3. Pituitary volume was 212 (160-324) mm^3^ in NPF, 223 (152-379) mm^3^ in ICI-HP, and 290 (153-417) mm^3^ in IHP, with no significant differences among the three groups (Figure 3A, p=0.68). Next, the width of the pituitary stalk in each group is shown in Figure 3B. The width of the pituitary stalk was 1.7 (1.5-2.0) mm in NPF and 1.9 (1.6-2.2) mm in IHP, whereas it was 2.4 (2.4-2.7) mm in ICI-HP and was significantly larger as compared to the other groups (p<0.01). An empty sella complicated two cases (9.1%) in NPF, one case (7.7%) in ICI-HP, and one case (5.9%) in IHP, with no difference among the three groups (p=0.93).

Pituitary findings and CRH loading test results in participants of IAD

**Figure 4:**
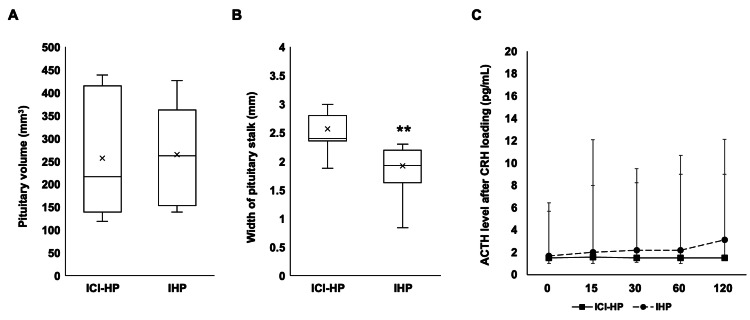
Pituitary findings on MRI in participants with IAD Pituitary findings on MRI in participants with immune checkpoint inhibitor-induced hypopituitarism (ICI-HP, n=11) and idiopathic hypopituitarism (IHP, n=9). (A) Pituitary volume (p=0.71, r=0.056). (B) Pituitary stalk width (p<0.01, r=-0.394). (C) Results of CRH loading tests in participants of ICI-HP and IHP. The Wilcoxon test was used for analysis. ** p<0.01.

In the present study, 11 of the ICI-HP participants and 9 of the IHP participants had IAD as their endocrine disruption pattern. MRI pituitary findings in cases of IAD in ICI-HP and IHP are shown in Figure 4. Pituitary volumes did not differ significantly among each group (p=0.68, Figure 4A). The width of the pituitary stalk was 1.9 (1.6-2.2) mm in IHP, and ICI-HP was 2.4 (2.4-2.8) mm, significantly thicker than the IHP group (p<0.01, Figure 4B). The results of the CRH loading test are then shown in Figure 4C. Adrenocorticotropic hormone (ACTH) levels were no significant differences between ICI-HP and IHP. In the IAD participants, the cortisol levels before CRH loading were 1.8 (0.3-5.5) μg/dL for ICI-HP, 2.0 (0.2-4.1) μg /dL, and NPF 11.3 (7.1-13.8) μg/dL. There was no significant difference in cortisol levels between participants in the ICI-HP and IHP groups (p=0.99).

## Discussion

This retrospective data revealed that enlarged pituitary stalks are characteristic findings on MRI in patients with hypopituitarism after ICI administration. Pituitary inflammation is an inflammation of the pituitary gland and is one of the classic manifestations of immune-related side effects. It causes headaches and secondary endocrinopathy, depending on the site and extent of damage caused by the pituitary infiltration of activated T cells [5]. Characteristic MRI findings have not been established for ICI-HP [9]. Although hypopituitarism by ICI is characterized by pituitary enlargement and enlarged pituitary stalk as imaging features, there are many cases without pituitary enlargement at the time of diagnosis [3]. However, in the present study, no pituitary enlargement was identified compared to controls, NPF, and IHP. Factors reported to influence pituitary size include age, gender, pregnancy, and history of oral contraceptive use [11,12]. The ICI-HP participants in this study were older than the NPF participants, which may be one reason for the lack of difference in pituitary size. The time of diagnosis of ICI-HP in the study participants was 125 (56-212) days after the first administration of ICI. A previous report reported that pituitary enlargement diagnosed with ICI-HP improves with time, as do signal changes within the pituitary gland on contrast-enhanced MRI, suggesting that the pituitary findings may change depending on the timing of the examination [8]. In addition, while most cases of CTLA-4 inhibitor-induced hypopituitarism have imaging findings at the time of diagnosis [13], patients who received CTLA-4 inhibitors were not included in this study. When a power analysis was performed in this study, the statistical power for detecting differences in enlarged pituitary stalks was 99.9%, with a minimum sample size of 12 cases required to achieve significance, suggesting that the observed results are robust. In contrast, the minimum sample size required to detect a significant difference in pituitary volume was 122 cases, indicating that the current study may lack sufficient sample size to detect a significant effect in this parameter. These factors may have led to differences in findings from previous reports.

Participants with ICI-HP in this study had the highest rate of presenting with IAD. IAD is the most common cause of hypoadrenocorticism due to ICI and the most frequent endocrine disorder in patients with ICI-HP [14]. Steroid therapy is an effective treatment for ICI-HP and ICI-related hypoadrenocorticism. Still, IAD symptoms may be masked and diagnosis delayed when steroid therapy is used in combination with treatment for malignancy [15]. If there are findings on MRI in cases of ICI and concomitant steroid therapy, it is advisable to consider the possibility of other hypopituitarism complications in the examination.

This study has several limitations. First, in this study, NPF, IHP, and ICI-HP had different patient backgrounds. The NPF participants were younger than the other groups, which may include selection bias. In addition, idiopathic hypopituitarism cases may also have diseases other than the apparent causes excluded in this study. Second, this is a single-center, retrospective observational study with Japanese patients only, and the results may not be applicable to all races, regions, etc. In addition, this study analyzed the findings of a single MRI scan performed at the time of diagnosis and did not consider changes in findings over time or findings on contrast-enhanced MRI scans. Finally, this study's small sample size of irAE cases precluded analysis stratified by concomitant anticancer agents, primary disease, or the presence or absence of radiation therapy. And, because of the small sample size, it was not possible to analyze confounding factors that could affect pituitary MRI findings. In order to resolve these statistical limitations, it will be necessary to accumulate further case data in the future.

## Conclusions

In conclusion, an enlarged pituitary stalk may be characteristic imaging findings in hypopituitarism due to ICI. Endocrine testing should be considered in subjects with an enlarged pituitary stalk on an MRI scan since the symptoms of hypopituitarism may be masked by steroid therapy, which was used as an anticancer drug or as a treatment for other irAEs.
